# Evaluation of Candidate Nephropathy Susceptibility Genes in a Genome-Wide Association Study of African American Diabetic Kidney Disease

**DOI:** 10.1371/journal.pone.0088273

**Published:** 2014-02-13

**Authors:** Nicholette D. Palmer, Maggie C. Y. Ng, Pamela J. Hicks, Poorva Mudgal, Carl D. Langefeld, Barry I. Freedman, Donald W. Bowden

**Affiliations:** 1 Department of Biochemistry, Wake Forest School of Medicine, Winston-Salem, North Carolina, United States of America; 2 Center for Genomics and Personalized Medicine Research, Wake Forest School of Medicine, Winston-Salem, North Carolina, United States of America; 3 Diabetes Research Center, Wake Forest School of Medicine, Winston-Salem, North Carolina, United States of America; 4 Center for Public Health Genomics and Department of Biostatistical Sciences, Wake Forest School of Medicine, Winston-Salem, North Carolina, United States of America; 5 Department of Internal Medicine, Wake Forest School of Medicine, Winston-Salem, North Carolina, United States of America; Mario Negri Institute for Pharmacological Research and Azienda Ospedaliera Ospedali Riuniti di Bergamo, Italy

## Abstract

Type 2 diabetes (T2D)-associated end-stage kidney disease (ESKD) is a complex disorder resulting from the combined influence of genetic and environmental factors. This study contains a comprehensive genetic analysis of putative nephropathy loci in 965 African American (AA) cases with T2D-ESKD and 1029 AA population-based controls extending prior findings. Analysis was based on 4,341 directly genotyped and imputed single nucleotide polymorphisms (SNPs) in 22 nephropathy candidate genes. After admixture adjustment and correction for multiple comparisons, 37 SNPs across eight loci were significantly associated (1.6E-05<P_emp_<0.049). Among these, variants in *MYH9* were the most significant (1.6E-05<P_emp_<0.049), followed by additional chromosome 22 loci (*APOL1*, *SFI1*, and *LIMK2*). Nominal signals were observed in *AGTR1*, *RPS12*, *CHN2* and *CNDP1*. Additional adjustment for *APOL1* G1/G2 risk variants attenuated association at *MYH9* (P_emp_ = 0.00026–0.043) while marginally improving significance of other *APOL1* SNPs (rs136161, rs713753, and rs767855; P_emp_ = 0.0060–0.037); association at other loci was markedly reduced except for *CHN2* (chimerin; rs17157914, P_emp_ = 0.029). In addition, SNPs in other candidate loci (*FRMD3* and *TRPC6*) trended toward association with T2D-ESKD (P_emp_<0.05). These results suggest that risk contributed by putative nephropathy genes is shared across populations of African and European ancestry.

## Introduction

Diabetes-associated kidney disease (DKD) is the most common cause of nephropathy in western societies, present in approximately 40% of patients with type 2 diabetes (T2D) [1]. Patients with DKD account for half of the incident cases of end-stage kidney disease (ESKD) in the United States. T2D-ESKD is a devastating complication with five year survivals in the range of 30% [2]. Current trends suggest that the prevalence of DKD will continue to increase [1] constituting a significant socioeconomic burden on the healthcare system and resulting in increased morbidity and mortality.

T2D-ESKD is a complex disorder resulting from genetic and environmental factors [reviewed in [3]]. Among population groups, African Americans have the highest incidence and prevalence and the rate of new ESKD cases has grown by 7.0% since 2000 [2]. In contrast to risk for non-diabetic ESKD in African Americans, which is powerfully associated with genetic variants in the apolipoprotein L1 gene (*APOL1*; [4]), evidence to date suggests that T2D-ESKD has multi-factorial genetic risk.

The purpose of this study was to extend analysis of data from a previous genome-wide association study (GWAS) of T2D-ESKD in the African American population [5] with detailed assessment of genes previously implicated in ESKD susceptibility while accounting for the effects observed at the *APOL1* locus. The value of this dataset has been enhanced through imputation of genotypes for over 2.2 million additional single nucleotide polymorphisms (SNPs) in the GWAS subjects which facilitates a comprehensive evaluation of putative susceptibility genes for association with T2D-ESKD in African Americans.

## Results

### Clinical Characteristics of Study Samples

The clinical characteristics of study participants included in the GWAS are shown in **Table 1**. T2D-ESKD cases tended to have a higher proportion of females (P = 0.076), possibly reflecting the increased prevalence of T2D among African American women [6], participation bias, and survival. In addition, the age at enrollment for T2D-ESKD subjects is older than that for the control groups (P<0.0001); however, the age at enrollment for the control groups is older than the age of T2D diagnosis in T2D-ESKD subjects (P<0.0001). Notably, the use of population-based controls has not precluded the identification of trait associations in other investigations (e.g. [7]). Both cases and controls were overweight or obese at the time of enrollment (P = 0.30).

**Table 1:** Clinical Characteristics of Study Participants.

	T2D-ESKD	Control	p-value
*N*	965	1029	
Female (%)	61.2%	57.3%	0.076
Age at Enrollment (years)	61.6±10.5	49.0±11.9	<0.0001
Age at T2D Diagnosis (years)	41.6±12.4	-	<0.0001*
Age at ESKD Diagnosis (years)	58.0±10.9	-	<0.0001*
T2D to ESKD Duration (years)	16.2±10.9	-	
Retinopathy (n)	507	0	
BMI (kg/m^2^)	29.7±7.0	30.0±7.0	0.30

* compared to control subjects.

### GWAS

A total of 832,357 directly genotyped autosomal SNPs passed quality control and were tested for association in 965 T2D-ESKD cases and 1029 controls lacking T2D and ESKD. Only a modest increase in the inflation factor was noted with inclusion of related individuals (1.04 versus 1.06) therefore, cryptic first degree relatives (n = 54) were retained in the analysis. In addition, >2.28 million SNPs were imputed from HapMap II release 22. Results from twenty-two T2D-DKD candidate loci with 10 kb flanking sequence upstream and downstream (n = 4333 SNPs; **Table S1**) were selected for subsequent analysis.

### T2D-ESKD Candidate Loci

We defined T2D-ESKD candidate loci as genes which have been implicated in ESKD (diabetes associated or non-diabetes associated) either through direct (e.g. *CNDP1*, *APOL1*) genetic analysis or through compelling functional relationships (e.g. *REN*). In addition, candidates highlighted from a previous genome-wide association study (GWAS) of T2D-ESKD in this African American population [5] were included (i.e. *RPS12*, *LIMK2*, and *SFI1*). Analysis of the twenty two loci (n = 4333 SNPs) using PC1 as a covariate revealed nominal association (P<0.05) at 20 loci (n = 382 SNPs; **Table S1 and Figure S1**). Correction for multiple comparisons using the number of independent tests at each locus identified eight loci (38 SNPs) with empiric p-values less than 0.05 (P_emp_<0.05; **Table 2**). The most significant signal (rs5750250, P_emp_ = 1.6E-05, OR (95% CI) = 0.72 (0.63–0.81)) was observed in intron 13 of the non-muscle myosin heavy chain 9 (*MYH9*) gene. Of the 157 SNPs evaluated in *MYH9* resulting in 56 independent SNPs, 12 SNPs had P_emp_<0.05 and were modestly correlated (0.02<r^2^<0.89; **Figure S2**). Association was also observed with three additional loci on chromosome 22: apolipoprotein L1 (*APOL1*; P_emp_ = 0.0068–0.045; 0.03<r^2^<0.82, **Figure S2**), LIM domain kinase 2 (*LIMK2*; P_emp_ = 0.020–0.024; r^2^>0.99, **Figure S 2**), and Sfi1 homolog (*SFI1*; P_emp_ = 0.044). Among other significant loci, two SNPs (rs9493454 and rs7769051; r^2^ = 0.67) in the ribosomal protein S12 (*RPS12*) gene were associated with T2D-ESKD (P_emp_ = 0.0071 and 0.0083, respectively). Imputation revealed two SNPs (rsq>0.90) in moderate LD (r^2^ = 0.49) located in the cytosol nonspecific dipeptidase 1 (*CNDP1*) gene which remained significant after correction for 45 effective SNPs tested (rs4892249, P_emp_ = 0.043 and rs6566815, P_emp_ = 0.0076). Additionally, nominal signals were observed at the angiotensin II receptor type 1 (*AGTR1*; P_emp_ = 0.018) and chimerin 2 (*CHN2*; P_emp_<0.044; 0.00<r^2^<0.05) genes.

**Table 2:** Association of Candidate Diabetic Kidney Disease Loci with T2D-ESKD.

SNP	Position^1^	Gene	Eff^2^	RA^3^	OR^4^	95% CI^5^	P_Add_ 6	P_emp_ 7
rs12695897	3:149927874	*AGTR1*	40	T	0.58	0.43–0.79	0.00046	0.018
rs9493454	6:133186322	*RPS12*	13	C	1.25	1.10–1.42	0.00055	0.0071
rs7769051	6:133188489	*RPS12*	13	A	1.26	1.10–1.44	0.00063	0.0083
rs3793313	7:29272298	*CHN2*	194	T	1.40	1.17–1.68	0.00023	0.044
rs2057737	7:29385620	*CHN2*	194	A	1.79	1.32–2.44	0.00020	0.038
rs3729621*	7:29487958	*CHN2*	194	G	1.57	1.24–1.99	0.00019	0.037
rs4892249*	18:70404575	*CNDP1*	45	G	1.25	1.09–1.42	0.00095	0.043
rs6566815*	18:70406087	*CNDP1*	45	T	1.31	1.14–1.51	0.00017	0.0076
rs2413035	22:29930460	*LIMK2*	20	T	0.60	0.44–0.82	0.0012	0.024
rs737888*	22:29941549	*LIMK2*	20	A	0.60	0.44–0.81	0.00099	0.020
rs5753521*	22:29968027	*LIMK2*	20	T	0.60	0.44–0.81	0.00099	0.020
rs2283879*	22:29968448	*LIMK2*	20	G	0.60	0.44–0.81	0.00099	0.020
rs2106294	22:29975759	*LIMK2*	20	C	0.60	0.44–0.81	0.00099	0.020
rs4820043	22:29977094	*LIMK2*	20	A	0.60	0.44–0.81	0.00099	0.020
rs2078803*	22:29982852	*LIMK2*	20	G	0.60	0.44–0.81	0.00099	0.020
rs2073857*	22:29988281	*LIMK2*	20	A	0.60	0.44–0.81	0.00099	0.020
rs737684*	22:29989827	*LIMK2*	20	C	0.60	0.44–0.81	0.00099	0.020
rs4141404*	22:30005185	*LIMK2*	20	A	0.60	0.44–0.81	0.00099	0.020
rs1858821*	22:30006454	*LIMK2*	20	T	0.60	0.44–0.81	0.00099	0.020
rs2040533*	22:30009110	*LIMK2*	20	G	0.60	0.44–0.81	0.00099	0.020
rs5753543*	22:30015458	*LIMK2*	20	G	0.60	0.44–0.81	0.0012	0.023
rs5749286	22:30230359	*SFI1*	27	A	0.62	0.46–0.84	0.0016	0.044
rs136161	22:34987378	*APOL1*	9	C	0.77	0.67–0.90	0.00076	0.0068
rs713753	22:34988480	*APOL1*	9	T	0.79	0.67–0.92	0.0032	0.029
rs767855*	22:35002241	*APOL1*	9	T	0.68	0.52–0.89	0.0050	0.045
rs735853	22:35009161	*MYH9*	56	G	0.68	0.55–0.84	0.00034	0.019
rs6000229	22:35016105	*MYH9*	56	T	0.75	0.66–0.86	2.3E-05	0.0013
rs2071730*	22:35019972	*MYH9*	56	C	0.79	0.69–0.91	0.00087	0.049
rs16996648	22:35022698	*MYH9*	56	C	1.29	1.12–1.48	0.00028	0.016
rs4821480*	22:35025193	*MYH9*	56	T	0.73	0.64–0.84	5.1E-06	0.00029
rs4821481*	22:35025888	*MYH9*	56	T	0.73	0.64–0.84	4.8E-06	0.00027
rs5750248*	22:35032838	*MYH9*	56	C	0.74	0.65–0.85	7.7E-06	0.00043
rs1557529*	22:35035475	*MYH9*	56	A	1.35	1.17–1.56	4.1E-05	0.0023
rs2157256	22:35037607	*MYH9*	56	G	0.80	0.70–0.91	0.00085	0.048
rs5750250	22:35038429	*MYH9*	56	A	0.72	0.63–0.81	2.9E-07	1.6E-05
rs5756152*	22:35042418	*MYH9*	56	A	1.39	1.17–1.64	0.00015	0.0086
rs2239784*	22:35044581	*MYH9*	56	C	0.73	0.61–0.87	0.00049	0.027

1 hg18;

2 Eff, effective number of SNPs per locus;

3 RA, reference allele;

4 OR, odds ratio;

5 95% CI, 95% confidence interval;

6 P_Add_, additive p-value;

7 P_emp_, empiric p-value;

* imputed SNPs.

### T2D-ESKD Candidate Loci with Adjustment for APOL1 G1 and G2 Variants

Adjustment for the *APOL1* G1 and G2 nephropathy risk variants [4] marginalized but did not abolish the significant evidence of associations observed at three loci (P_emp_<0.048; n = 5 SNPs; **Table 3**). The strongest signal observed was located downstream of the *CNDP1* gene (rs6566815, P_emp_ = 0.011). Three signals of association were observed at the *CHN2* locus (rs17157914, P_emp_ = 0.026; rs3793313, P_emp_ = 0.043; rs17157908, P_emp_ = 0.048). Two of these signals, which were highly correlated (rs17157908 and rs17157914, r^2^ = 0.94), emerged only after adjustment for *APOL1* G1/G2 (rs17157914, P_emp_ = 0.091 and rs17157908, P_emp_ = 0.14 prior to *APOL1* G1/G2 adjustment) while the initial signal at rs3793313 maintained the same level of significance (P_emp_ = 0.043). The previous single signal of association observed in the *AGTR1* gene remained significant (rs12695897, P_emp_ = 0.032) after accounting for the effects at the *APOL1* locus. In contrast, the previously observed significant association in the *MYH9* gene was abolished (rs5750250, P_emp_ = 0.20) as were other signals observed on chromosome 22 (P_emp_>0.099).

**Table 3:** Association of Candidate Diabetic Kidney Disease Loci with T2D-ESKD after Adjustment for *APOL1* G1/G2.

					Covariates: PC1				Covariates: PC1 and *APOL1* G1/G2			
SNP	Position^1^	Gene	Eff^2^	RA^3^	OR^4^	95% CI^5^	P_Add_ 6	P_emp_ 7	OR^4^	95% CI^5^	P_Add_ 6	P_emp_ 7
rs12695897	3:149927874	*AGTR1*	40	T	0.58	(0.43–0.79)	4.6E-04	0.018	0.58	(0.43–0.80)	7.9E-04	0.032
rs3793313	7:29272298	*CHN2*	194	T	1.40	(1.17–1.68)	4.6E-04	0.044	1.43	(1.18–1.72)	2.2E-04	0.043
rs17157908	7:29413882	*CHN2*	194	C	0.70	(0.57–0.86)	7.3E-04	0.14	0.66	(0.53–0.83)	2.5E-04	0.048
rs17157914	7:29414945	*CHN2*	194	A	0.69	(0.56–0.85)	4.7E-04	0.091	0.65	(0.52–0.81)	1.3E-04	0.026
rs6566815*	18:70406087	*CNDP1*	45	T	1.31	(1.14–1.51)	1.6E-04	0.076	1.32	(1.14–1.53)	2.5E-04	0.011

1 hg18;

2 Eff, effective number of SNPs per locus;

3 RA, reference allele;

4 OR, odds ratio;

5 95% CI, 95% confidence interval;

6 P_Add_, additive p-value;

7 P_emp_, empiric p-value;

* imputed SNP.

## Discussion

The goal of this study was to perform a detailed genetic analysis of reported ESKD susceptibility genes in a large African American cohort. Previous studies have been few in number and limited in scope focusing on divergent populations and evaluating relatively few variants by modern day standards. Advantages of this study include a comprehensive evaluation of genetic variation at each susceptibility locus using directly genotyped and imputed SNPs in analysis. In addition, this study uses a single population in which to compare and contrast findings from all reported loci.

After correction for the effective number of variants tested at each locus (**Table 2**), we identified eight susceptibility loci as nominally associated with T2D-ESKD. Examination of the risk allele burden of these variants (n = 37) in the eight loci revealed an increased risk allele burden (P<0.0001) with cases, on average, carrying 50.2 risk alleles while controls carried 47.0 (data not shown). The most significant signal was observed at the *MYH9* locus (rs5750250, P_emp_ = 1.6E-05) although this signal was abolished (P_emp_ = 0.20) after adjustment for the *APOL1* G1/G2 risk alleles. While this finding could be attributed to the potential inclusion of non-diabetic ESKD cases samples, the vast majority (>74%) of the case population had a duration of T2D greater than 5 years before initiating renal replacement therapy. Notably, this variant was the most significant SNP from our T2D-ESKD GWAS [5] despite inclusion of additional imputed variants to increase coverage in the current study, i.e. GWAS coverage of *MYH9* with an r^2^>0.73 with 46 of 166 SNPs versus GWAS and imputed data coverage of *MYH9* with an r^2^>0.99 with 156 of 166 SNPs. SNP rs5750250 lies within the genomic interval spanned by the four SNPs (rs4821480, rs2032487, rs4821481, and rs3752462) composing the E1 risk haplotype [8] however, individually none of these variants were associated with T2D-ESKD (P_emp_>0.53). Interestingly, evidence of association at the *APOL1* locus was reduced in comparison to *MYH9* (P_emp_<0.045) despite impressive associations previously observed with kidney disease in African Americans [4]. These results suggest that kidney disease, in general, is a heterogeneous class of diseases, consistent with the observation of differential odds ratios (ORs) reported for *APOL1* in HIV-associated nephropathy (OR = 29), focal segmental glomerulosclerosis (FSGS; OR = 17) and hypertension-attributed end-stage kidney disease (H-ESKD; OR = 7.3) [4], [9].

The most significant signal of association observed after adjustment for the *APOL1* G1/G2 variants was rs6566815 (P_emp_ = 0.011; **Table 3**) which lies 2.8 kb downstream of the *CNDP1* locus on chromosome 18. This region has been linked to DKD in multiple populations [10], [11], [12], and *CNDP1* was later implicated as the basis of the linkage peak [13]. In addition, a single variant on chromosomes 3 in the *AGTR1* gene was observed to be associated with T2D-ESKD (rs12695897, P_emp_ = 0.032). The angiotensin II receptor type 1 gene product is an interesting biological candidate involved in the renin-angiotensin system (reviewed in [14]). Among 98 genotyped and imputed SNPs tested (40 effective tests), only the imputed SNP rs12695897 remained nominally associated. rs12695897 was a low frequency variant (MAF = 0.05) with good imputation quality (rsq = 0.95) which would have been missed without imputation since it was omitted from current GWAS arrays. The remaining three signals that survived multiple comparisons correction and adjustment for *APOL1* were located in the *CHN2* gene. The initial variant (rs3793313) observed in the PC-adjusted analysis remained significant and two additional correlated variants (rs17157908 and rs17157914) increased in significance. This locus has been reported to be modestly associated with DKD in European-derived studies of type 1 diabetes [15] however, their seminal SNP rs39059 was not associated in our analysis (P_emp_ = 0.61).

Despite improvements in coverage afforded through imputation, this GWAS is not without limitations. A primary limitation related to study design is the inclusion of 965 T2D-ESKD cases and 1029 population-based controls. While it is possible that T2D variants would be identified, in practice this has not been the case [5], [16]. This study had >80% power to detect common variants (MAF>0.10) with a detectable OR of 1.33, consistent with common disease, when considering a significance threshold of 0.05 (**Table S2**). An advantage of our study design is the focus on loci with a priori evidence of association thus limiting the need for more stringent genome-wide significance thresholds. Although this study included evaluation of putative T2D-ESKD susceptibility loci that were identified within the same dataset, the current analyses extend those findings by increasing coverage and as was observed with *CHN2*, increased the number of variants observed with association. Related to the analytical approach, the current analysis is derived from imputation using the HapMap Phase II reference panel. Recent technological advancements have lead the way for development of more superior reference panels that allow for a more comprehensive imputation of variants across the allele spectrum. As such, these additional low frequency variants may contribute to the underlying genetic architecture of disease and deserve further evaluation in larger sample sets that are more adequately powered to examine their contribution.

In conclusion, we performed a detailed genetic analysis of T2D-ESKD susceptibility genes identified from literature searches in a large African American cohort. This study demonstrates the need for more comprehensive genotyping arrays, especially in the African American population, and confirms the utility of imputation to increase coverage in existing datasets. These findings support the hypothesis that genetic variation contributes to risk of T2D-ESKD through the combined impact of multiple genetic variants contributing modest individual risk.

## Methods

### Ethics Statement

Recruitment and sample collection procedures were approved by the Institutional Review Board at Wake Forest School of Medicine. Written informed consent was obtained from all study participants.

### Clinical Characteristics of Study Samples

This cross-sectional case-control study was designed to examine the genetics of T2D and ESKD in African Americans [5]. Patients with T2D-ESKD were recruited from dialysis facilities. T2D was diagnosed in African Americans who reported developing T2D after the age of 25 years and did not receive only insulin therapy since diagnosis. In addition, cases had at least one of the following three criteria for inclusion: a) T2D diagnosed at least 5 years before initiating renal replacement therapy, b) background or greater diabetic retinopathy by self-report and/or c) ≥100 mg/dl proteinuria on urinalysis in the absence of other causes of nephropathy. Unrelated African American controls without a current diagnosis of diabetes or renal disease were recruited from the community and internal medicine clinics. All T2D-ESKD cases and controls lacking T2D and ESKD were born in North Carolina, South Carolina, Georgia, Tennessee or Virginia. DNA extraction was performed using the PureGene system (Gentra Systems; Minneapolis, MN).

### Genotyping, Imputation, and Variant Selection

As reported previously [5], genotyping was performed on the Affymetrix Genome-wide Human SNP array 6.0 (Affy6.0). Genotypes were called using Birdseed version 2; APT 1.10.0 by grouping samples by DNA plate to determine the genotype cluster boundaries. All autosomal SNPs (n = 868,157) were included in analysis. Imputation was performed for autosomes using MACH (version 1.0.16, http://www.sph.umich.edu/csg/abecasis/MaCH/). SNPs with minor allele frequency (MAF) ≥1%, call rate ≥95% and Hardy–Weinberg p-value≥10^−4^ were used for imputation. A 1∶1 mixture of the HapMap II release 22 (NCBI build 36) CEU:YRI consensus haplotypes (http://mathgen.stats.ox.ac.uk/impute/) was used as a reference panel. Imputation was performed in two steps. For the first step, 484 unrelated African American samples were randomly selected to calculate recombination and error rate estimates. In the second step, these rates were used to impute all samples across the SNPs in the entire reference panel. Imputation results were filtered at an rsq threshold of ≥0.3 and a MAF ≥0.05. Adjustment for the *APOL1* G1/G2 risk variants was performed by direct genotyping of two SNPs in the *APOL1* G1 nephropathy risk variant (rs73885319; rs60910145) and an indel for the G2 risk variant (rs71785313) [4] on the Sequenom platform (San Diego, CA).

SNPs in twenty two genes with prior evidence of association with nephropathy were examined. These included genes located within linkage peaks from family-based linkage analysis (*NPHS1/2*, *TRPC6* and *CNDP1*/*2*), candidate gene studies (*AGTR1, NOS3*, *ACACB* and *ACTN4*), gene expression studies (*PLCE1*), admixture mapping (*APOL1* and *MYH9*), and GWAS (*CPVL*, *CHN2*, *ELMO1*, *FRMD3*, *CARS*, *PVT1*, and *ACE*) including candidates highlighted from a previous genome-wide association study (GWAS) of T2D-ESKD in this African American population [5] (i.e. *RPS12*, *LIMK2*, and *SFI1*). (**Table S1**).

### Statistical Analysis

#### Principal Component Analysis (PCA)

To address the effect of admixture in this African American dataset we performed a Principal Components Analysis (PCA) using all GWAS SNPs that passed quality control, excluding regions of high linkage disequilibrium (LD) and inversions. This approach was an iterative process whereby all high quality autosomal SNPs were used to calculate the top 50 principal components. Once calculated, the principal components were examined to determine if they were narrowly associated to specific regions of the genome. If so, those SNPs were excluded and the analysis repeated. The first principal component (PC1) explained the largest proportion of variation at 22% and was used as a covariate in all analyses. A direct comparison of the PCA with FRAPPE [17] analysis of 70 ancestry informative markers (AIMs; [18]) resulted in a high correlation between PC1 and the AIMs ancestry estimates, r^2^ = 0.87. The mean (SD) African ancestry proportion in 965 T2D-ESKD cases and 1,029 controls was 0.80±0.11 and 0.78±0.11, respectively, as estimated by FRAPPE analysis. The remaining principal components explained markedly less variation and were associated with specific regions of the genome not in proximity to the candidate genes of interest; thus, not relecting either global or local ancestry [19]. Therefore, PC1 was used as a covariate in the association analyses to adjust for population substructure.

#### Locus-specific Analysis

Twenty-two previously reported T2D-DKD susceptibility loci were evaluated (**Table S1**). For regional analysis, each gene was defined as +/−10 kb from the longest annotated transcript. To test for an association with T2D-ESKD a logistic regression model was computed adjusting for PC1 and assuming an additive genetic model for each SNP individually using SNPGWA (www.phs.wfubmc.edu; [20]) with adjustment for PC1. In addition, variants were tested for association after adjusting for the influence of the *APOL1* G1/G2 risk variants. Based on the known linkage disequilibrium pattern where G1 and G2 risk variants are very rarely observed on a single chromosome, we constructed a binary variable representing the compound G1/G2 risk across these three markers, modeling *APOL1* risk for all individuals with recessive haplotypes at either G1 or G2 or heterozygosity at both G1 and G2. *APOL1* has been shown to be powerfully associated with non-diabetic ESKD [4] and has been demonstrated to confound evidence of association in T2D-ESKD [21]. A Bonferroni *P* value (P_emp_) <0.05 corrected for the effective number of SNPs at each locus, i.e. independent SNPs in each locus was counted using the Li and Ji method [22] implemented in Sequential Oligogenic Linkage Analysis Routines (SOLAR), was considered statistically significant. Each locus was evaluated for the effective number of SNPs under the assumption of distinct pathways ultimately contributing to the overarching disease examined herein, diabetic nephropathy.

#### Power

The power of the tests of association were calculated using the genetic power calculator [23]. Estimates were based on a a sample of 965 T2D-ESKD cases and 1029 controls lacking T2D and ESKD under and additive genetic model assuming and r^2^ = 1 of the genotyped variant with the causal variant for a disease prevalence of T2D-ESKD estimated at 1% in the African American population [24].

## Supporting Information

Figure S1 **Regional association plots for proposed T2D-ESRD susceptibility loci.**
(DOCX)

Figure S2 **LD Structure among associated T2D-ESRD susceptibility loci.**
(DOCX)

Table S1 **Proposed T2D-ESRD susceptibility loci identified for follow-up study in the African American T2D-ESRD cohort.**
(DOCX)

Table S2 **Power calculations for a sample of 965 T2D-ESKD cases and 1029 controls lacking T2D and ESKD under and additive genetic model assuming and r^2^ = 1 of the genotyped variant with the causal variant for a disease prevalence of T2D-ESKD estimated at 1%.**
(DOCX)
