# Supplementary material for: Evaluation of Candidate Nephropathy Susceptibility Genes in a Genome-Wide Association Study of African American Diabetic Kidney Disease
Source: PLoS One. 2014 Feb 13;9(2):e88273. doi: 10.1371/journal.pone.0088273 (PMC3923777; doi:10.1371/journal.pone.0088273)
Supplement: Figure S1 — Regional association plots for proposed T2D-ESRD susceptibility loci. (DOCX) [file pone.0088273.s001.docx]

**Figure S1. Regional association plots for proposed T2D-ESRD susceptibility loci.** The variant most robustly associated is denoted in purple annotated by SNP ID with additional genotyped and imputed SNPs passing quality controls. SNPs are plotted with their p-values as a function of position (hg19). Shape of the data point is indicative of function, i.e. non-synonymous (▼) and no annotation (●), and color indicates LD (r^2^) with the previously identified variant taken from HapMap (red, r^2^=0.8-1.0; yellow, r^2^=0.6-0.8; green, r^2^=0.4-0.6; cyan, r^2^=0.2-0.4, and blue, r^2^<0.2). Estimated recombination rates (HapMap) reflect the local LD structure. Gene annotations were taken from the University of California Santa Cruz genome browser.

**
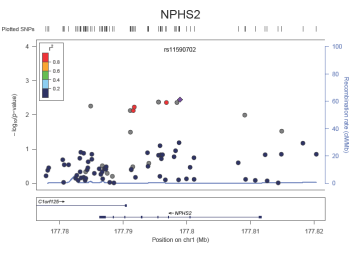

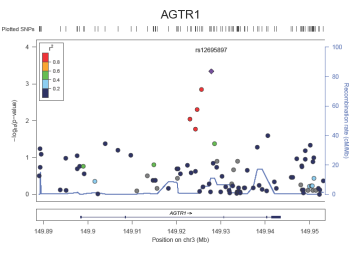
A. B.**

**
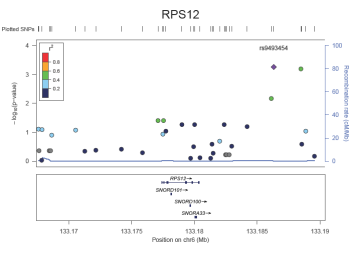

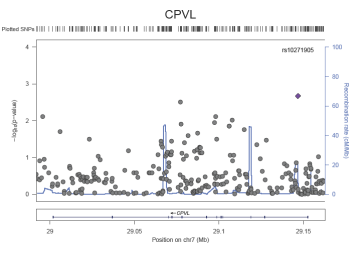
C. D.**

**
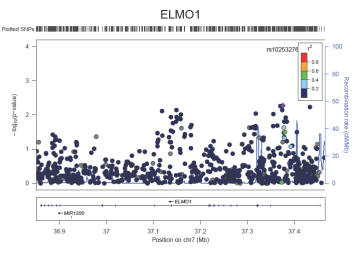
E.
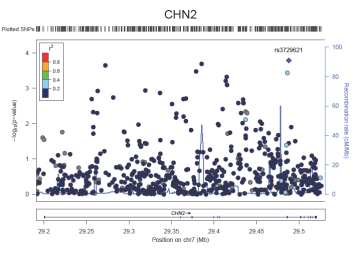
 F.**

**G
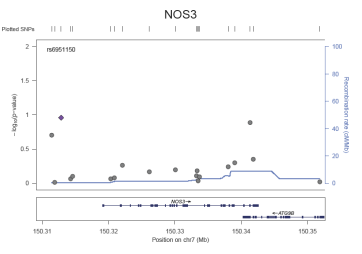
 H.
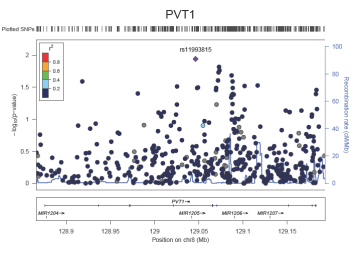
**

**
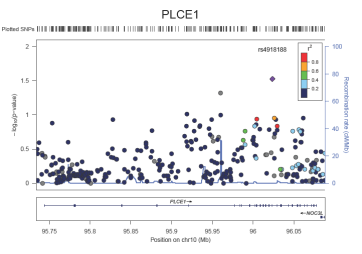
I.
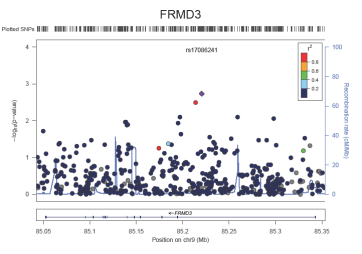
 J.**

**
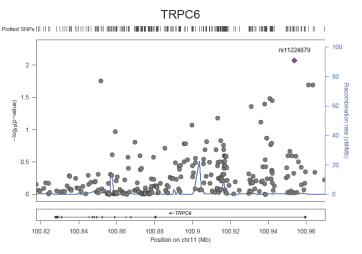
K.
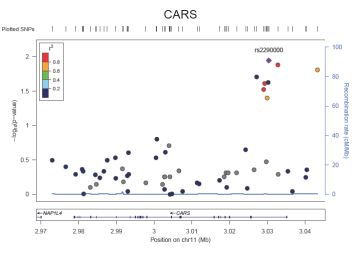
 L.**

**
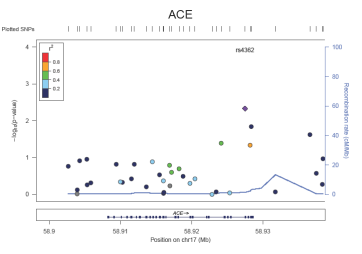
M.
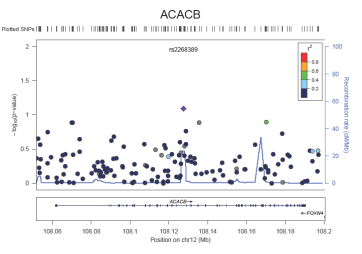
 N.**

**
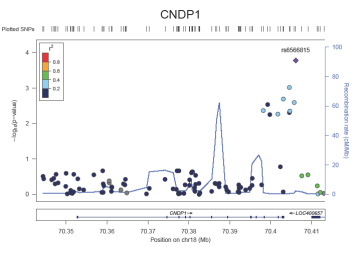
O.
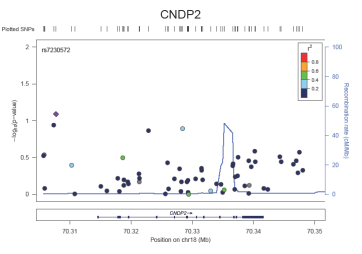
 P.**

**Q.
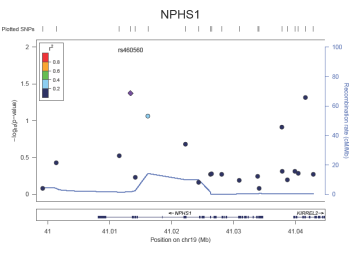
 R.
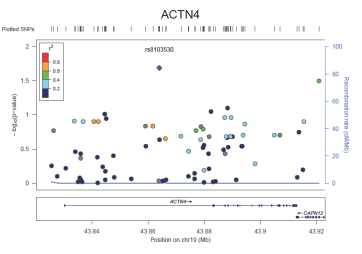
**

**
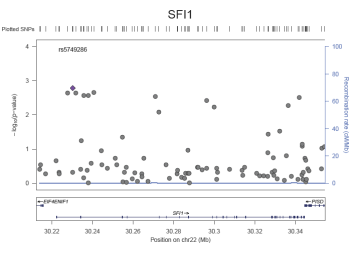
S.
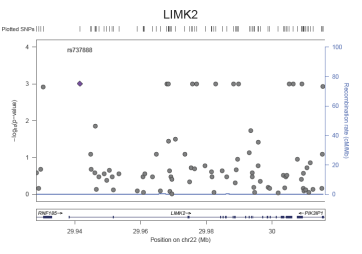
 T.**

**
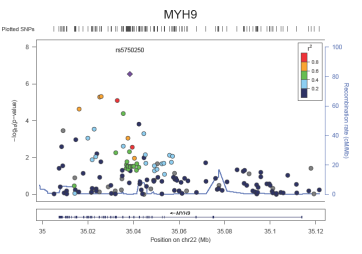
U.
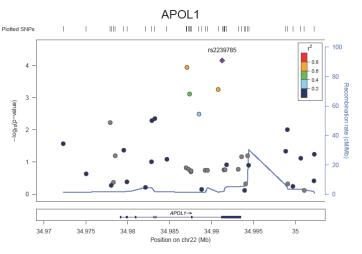
 V.**
