# Supplementary material for: Evaluation of Candidate Nephropathy Susceptibility Genes in a Genome-Wide Association Study of African American Diabetic Kidney Disease
Source: PLoS One. 2014 Feb 13;9(2):e88273. doi: 10.1371/journal.pone.0088273 (PMC3923777; doi:10.1371/journal.pone.0088273)
Supplement: Figure S2 — LD Structure among associated T2D-ESRD susceptibility loci. (DOCX) [file pone.0088273.s002.docx]

**Figure S2. LD Structure among associated T2D-ESRD susceptibility loci.** Haploview-generated LD map in unrelated African-American controls (n=988). SNPs are ordered by position with rs# and gene annotations. The gradient color of each box indicates the D’ between intersecting SNPs (red = high D’, white = low D’) and numbers within each box indicate the r^2^ value.


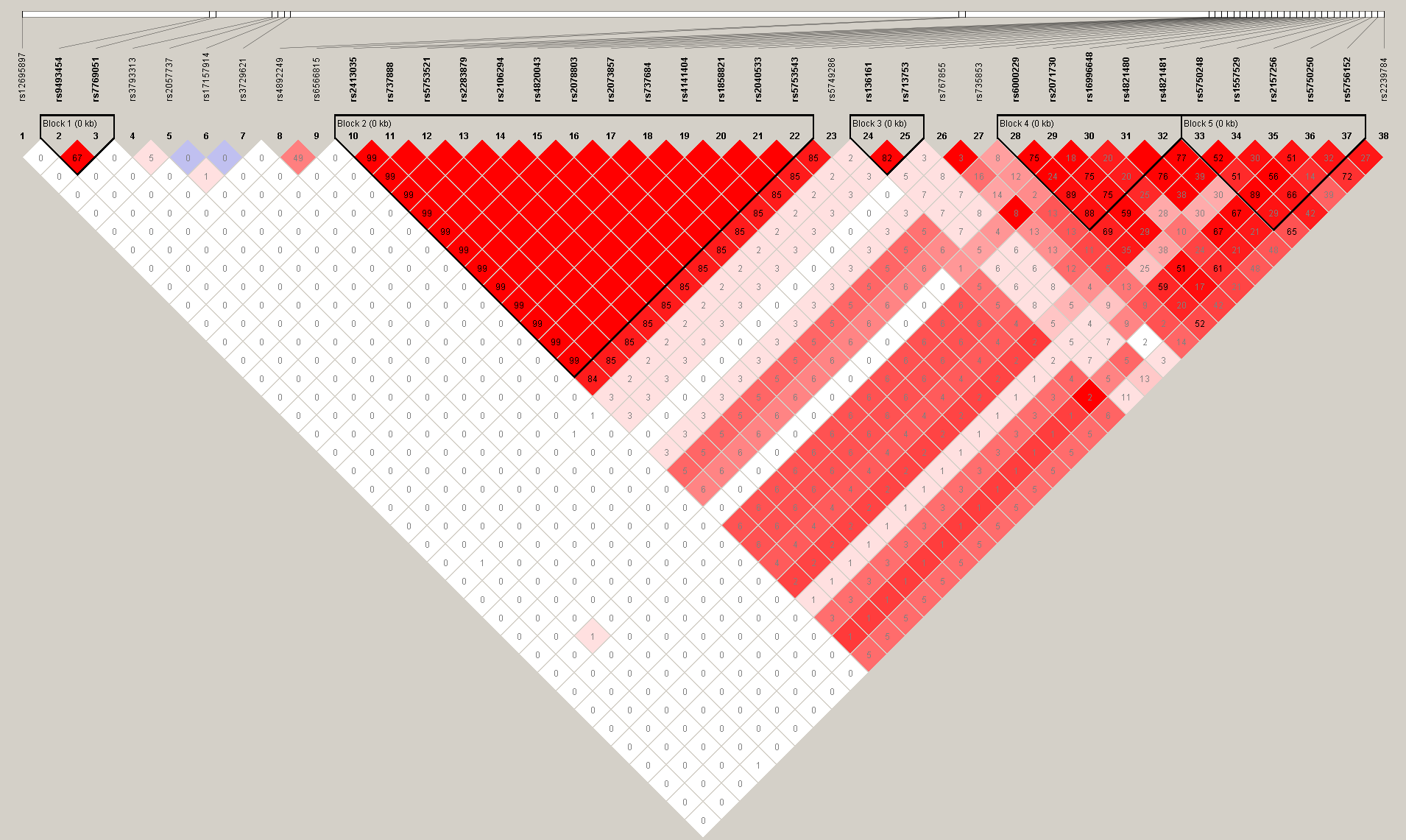


*AGTR1*

*RPS12*

*CHN2*

*CNDP1*

*LIMK2*

*SFI1*

*APOL1*

*MYH9*
