# Supplementary material for: Evaluation of Candidate Nephropathy Susceptibility Genes in a Genome-Wide Association Study of African American Diabetic Kidney Disease
Source: PLoS One. 2014 Feb 13;9(2):e88273. doi: 10.1371/journal.pone.0088273 (PMC3923777; doi:10.1371/journal.pone.0088273)
Supplement: Table S1 — Proposed T2D-ESRD susceptibility loci identified for follow-up study in the African American T2D-ESRD cohort. (DOCX) [file pone.0088273.s003.docx]

**Table S1.** **Proposed T2D-ESRD susceptibility loci identified for follow-up study in the African American T2D-ESRD cohort.**

| **Identifier** | **Gene Name** | **Position (hg18)** | **Reference** |
| --- | --- | --- | --- |
| *NPHS2* | Podocin | 1:177786297-177811707 | [[1](#_ENREF_1)] |
| *REN* | Renin | 1:204123944-204135465 | [[2](#_ENREF_2)] |
| *AGTR1* | Angiotensin II Receptor Type 1 | 3:149898348-149943480 | [[3](#_ENREF_3)] |
| *RPS12* | Ribosomal Protein S12 | 6:133177401-133180396 | [[4](#_ENREF_4)] |
| *CPVL* | Carboxypeptidase, vitellogenic-like | 7:29001772-29201417 | [[5](#_ENREF_5)] |
| *CHN2* | Chimerin 2 | 7:29200646-29520469 | [[5](#_ENREF_5)] |
| *ELMO1* | Engulfment and Cell Motility 1 | 7:36860486-37455036 | [[5-7](#_ENREF_5)] |
| *NOS3* | Nitric Oxide Synthase 3 | 7:150319080-150342609 | [[8](#_ENREF_8)] |
| *PVT1* | Plasmacytoma Variant Translocation 1 | 8:128875961-129182681 | [[9](#_ENREF_9)] |
| *FRMD3* | FERM Domain Containing 3 | 9:85047725-85343168 | [[5](#_ENREF_5)] |
| *PLCE1* | Phospolipase C, Epsilon 1 | 10:95743736-96078138 | [[10](#_ENREF_10)] |
| *CARS* | Cysteinyl-tRNA Synthetase | 11:2978735-3035247 | [[5](#_ENREF_5)] |
| *TRPC6* | Transient Receptor Potential Cation Channel | 11:100827505-100959869 | [[11](#_ENREF_11)] |
| *ACACB* | Acetyl-Coenzyme A Carboxylase Beta | 12:108038783-108190413 | [[12](#_ENREF_12), [13](#_ENREF_13)] |
| *ACE* | Angiotensin I Converting Enzyme | 17:58908166-58952935 | [[14](#_ENREF_14)] |
| *CNDP2* | Cytosol Nonspecific Dipeptidase 2 | 18:70314577-70339336 | [[15](#_ENREF_15)] |
| *CNDP1* | Cytosol Nonspecific Dipeptidase 1 | 18:70352672-70403241 | [[15](#_ENREF_15), [16](#_ENREF_16)] |
| *NPHS1* | Nephrin | 19:41008114-41034579 | [[1](#_ENREF_1)] |
| *ACTN4* | Alpha Actinin 4 | 19:43830167-43913010 | [[17](#_ENREF_17)] |
| *LIMK2* | LIM Domain Kinase 2 | 22:29938250-30006066 | [[4](#_ENREF_4)] |
| *SFI1* | Sfi1 Homolog | 22:30222261-30344534 | [[4](#_ENREF_4)] |
| *APOL1* | Apolipoprotein L1 | 22:34979070-34993523 | [[18](#_ENREF_18)] |
| *MYH9* | Non-muscle Myosin Heavy Chain 9 | 22:35007272-35113927 | [[19](#_ENREF_19), [20](#_ENREF_20)] |

**References**

1. Iyengar, S.K., et al., *Linkage analysis of candidate loci for end-stage renal disease due to diabetic nephropathy.* J Am Soc Nephrol, 2003. **14**(7 Suppl 2): p. S195-201.

2. Prasad, P., et al., *Chronic renal insufficiency among Asian Indians with type 2 diabetes: I. Role of RAAS gene polymorphisms.* BMC Med Genet, 2006. **7**: p. 42.

3. Doria, A., et al., *Synergistic effect of angiotensin II type 1 receptor genotype and poor glycaemic control on risk of nephropathy in IDDM.* Diabetologia, 1997. **40**(11): p. 1293-9.

4. McDonough, C.W., et al., *A genome-wide association study for diabetic nephropathy genes in African Americans.* Kidney Int, 2011. **79**(5): p. 563-72.

5. Pezzolesi, M.G., et al., *Genome-wide association scan for diabetic nephropathy susceptibility genes in type 1 diabetes.* Diabetes, 2009. **58**(6): p. 1403-10.

6. Leak, T.S., et al., *Variants in intron 13 of the ELMO1 gene are associated with diabetic nephropathy in African Americans.* Ann Hum Genet, 2009. **73**(2): p. 152-9.

7. Shimazaki, A., et al., *Genetic variations in the gene encoding ELMO1 are associated with susceptibility to diabetic nephropathy.* Diabetes, 2005. **54**(4): p. 1171-8.

8. Freedman, B.I., et al., *Genetic analysis of nitric oxide and endothelin in end-stage renal disease.* Nephrol Dial Transplant, 2000. **15**(11): p. 1794-800.

9. Hanson, R.L., et al., *Identification of PVT1 as a candidate gene for end-stage renal disease in type 2 diabetes using a pooling-based genome-wide single nucleotide polymorphism association study.* Diabetes, 2007. **56**(4): p. 975-83.

10. Lindenmeyer, M.T., et al., *Systematic analysis of a novel human renal glomerulus-enriched gene expression dataset.* PLoS One, 2010. **5**(7): p. e11545.

11. Winn, M.P., et al., *A mutation in the TRPC6 cation channel causes familial focal segmental glomerulosclerosis.* Science, 2005. **308**(5729): p. 1801-4.

12. Tang, S.C., et al., *The acetyl-coenzyme A carboxylase beta (ACACB) gene is associated with nephropathy in Chinese patients with type 2 diabetes.* Nephrol Dial Transplant, 2010. **25**(12): p. 3931-4.

13. Maeda, S., et al., *A single nucleotide polymorphism within the acetyl-coenzyme A carboxylase beta gene is associated with proteinuria in patients with type 2 diabetes.* PLoS Genet, 2010. **6**(2): p. e1000842.

14. Wang, F., et al., *Association between genetic polymorphism of the angiotensin-converting enzyme and diabetic nephropathy: a meta-analysis comprising 26,580 subjects.* J Renin Angiotensin Aldosterone Syst, 2012. **13**(1): p. 161-74.

15. Ahluwalia, T.S., E. Lindholm, and L.C. Groop, *Common variants in CNDP1 and CNDP2, and risk of nephropathy in type 2 diabetes.* Diabetologia, 2011. **54**(9): p. 2295-302.

16. Janssen, B., et al., *Carnosine as a protective factor in diabetic nephropathy: association with a leucine repeat of the carnosinase gene CNDP1.* Diabetes, 2005. **54**(8): p. 2320-7.

17. Bostrom, M.A., et al., *Relevance of the ACTN4 Gene in African-Americans with Non-Diabetic End-Stage Renal Disease.* Am J Nephrol, 2012. **36**(3): p. 252-260.

18. Genovese, G., et al., *Association of trypanolytic ApoL1 variants with kidney disease in African Americans.* Science, 2010. **329**(5993): p. 841-5.

19. Kopp, J.B., et al., *MYH9 is a major-effect risk gene for focal segmental glomerulosclerosis.* Nat Genet, 2008. **40**(10): p. 1175-84.

20. Kao, W.H., et al., *MYH9 is associated with nondiabetic end-stage renal disease in African Americans.* Nat Genet, 2008. **40**(10): p. 1185-92.
