# Supplementary material for: Evaluation of Candidate Nephropathy Susceptibility Genes in a Genome-Wide Association Study of African American Diabetic Kidney Disease
Source: PLoS One. 2014 Feb 13;9(2):e88273. doi: 10.1371/journal.pone.0088273 (PMC3923777; doi:10.1371/journal.pone.0088273)
Supplement: Table S2 — Power calculations for a sample of 965 T2D-ESKD cases and 1029 controls lacking T2D and ESKD under and additive genetic model assuming and r2 = 1 of the genotyped variant with the causal variant for a disease prevalence of T2D-ESKD estimated at 1%. (DOCX) [file pone.0088273.s004.docx]

**Table S2. Power calculations for a sample of 965 T2D-ESKD cases and 1029 controls lacking T2D and ESKD under and additive genetic model assuming and r^2^=1 of the genotyped variant with the causal variant for a disease prevalence of T2D-ESKD estimated at 1%.**

|  |  | Detectable Odds Ratio (Additive Model) | | | | | | |
| --- | --- | --- | --- | --- | --- | --- | --- | --- |
| Type I Error | Power | q=0.01 | q=0.05 | q=0.10 | q=0.20 | q=0.30 | q=0.40 | q=0.50 |
| 0.05 | 0.70 | 1.95 | 1.40 | 1.29 | 1.22 | 1.19 | 1.18 | 1.19 |
|  | 0.80 | 2.09 | 1.46 | 1.33 | 1.25 | 1.22 | 1.21 | 1.21 |
|  | 0.90 | 2.29 | 1.54 | 1.39 | 1.29 | 1.26 | 1.25 | 1.25 |
| 0.001 | 0.70 | 2.62 | 1.65 | 1.47 | 1.36 | 1.32 | 1.31 | 1.31 |
|  | 0.80 | 2.78 | 1.72 | 1.52 | 1.39 | 1.35 | 1.34 | 1.35 |
|  | 0.90 | 3.03 | 1.81 | 1.58 | 1.44 | 1.40 | 1.39 | 1.40 |
| 1.0E-05 | 0.70 | 3.30 | 1.90 | 1.64 | 1.49 | 1.44 | 1.43 | 1.45 |
|  | 0.80 | 3.48 | 1.97 | 1.69 | 1.53 | 1.48 | 1.47 | 1.49 |
|  | 0.90 | 3.75 | 2.07 | 1.76 | 1.58 | 1.53 | 1.52 | 1.55 |
| 2.5E-06 | 0.70 | 3.49 | 1.97 | 1.69 | 1.52 | 1.47 | 1.46 | 1.48 |
|  | 0.80 | 3.68 | 2.04 | 1.74 | 1.57 | 1.51 | 1.50 | 1.53 |
|  | 0.90 | 3.95 | 2.14 | 1.81 | 1.62 | 1.57 | 1.56 | 1.59 |
| 5.0E-08 | 0.70 | 4.01 | 2.14 | 1.81 | 1.62 | 1.57 | 1.56 | 1.59 |
|  | 0.80 | 4.23 | 2.22 | 1.87 | 1.67 | 1.61 | 1.60 | 1.64 |
|  | 0.90 | 4.50 | 2.33 | 1.95 | 1.73 | 1.67 | 1.67 | 1.72 |
